# *ELOVL*5 Regulates Ferroptosis in Breast Cancer Cells

**DOI:** 10.1134/S1607672925601246

**Published:** 2025-12-02

**Authors:** K. V. Klycheva, A. V. Razumovskaya, A. D. Shatsillo, M. D. Mastykina, T. A. Kulagin, M. O. Silkina, S. V. Nikulin

**Affiliations:** Faculty of Biology and Biotechnology, National Research University “Higher School of Economics”, Moscow, Russia

**Keywords:** breast cancer, *ELOVL*5, ferroptosis, erastin, docosahexaenoic acid

## Abstract

Today, breast cancer (BC) occupies a leading position in prevalence and mortality from oncological diseases among the female population worldwide. Ferroptosis is a special type of cell death associated with peroxidation of intracellular lipids. It is a promising option for the therapy of BC resistant to traditional methods of treatment. The *ELOVL*5 gene, involved in the elongation of long-chain polyunsaturated fatty acids (LC-PUFA), was previously associated with BC progression. In this work, the effect of *ELOVL*5 knockdown on the dynamics of ferroptosis induction in MDA-MB-231 cells under the influence of docosahexaenoic acid (DHA) and erastin was investigated. A comparative analysis of changes in the expression of individual genes under the influence of these agents was also carried out. It was shown that a decrease in *ELOVL*5 expression increases cell sensitivity to both agents, while DHA causes earlier cell death. The protective effect of ferroptosis inhibitors (ferrostatin-1 and deferoxamine) confirmed the involvement of this pathway in the observed effects. Differences in the expression of genes associated with oxidative stress, inflammation and proliferation were also revealed, indicating different molecular trajectories of ferroptosis in cells with different *ELOVL*5 gene expression. Thus, the present study deepens the understanding of the contribution of the *ELOVL*5 gene to the regulation of ferroptosis and can be used in the development of targeted therapy for breast cancer.

## INTRODUCTION

Breast cancer (BC) is one of the most common oncological diseases in women both in Russia and worldwide, remaining the main cause of death in this group [1]. According to 2022 data, BC accounted for more than 2.3 million new diagnoses and 670 000 registered deaths [2]. Despite advances in diagnosis and treatment, breast cancer remains one of the most aggressive forms of malignant tumors, characterized by a high risk of recurrence, which develops in approximately 40% of patients [3]. In addition, according to forecasts, the number of new cases and deaths is expected to increase by 2050 by 38 and 68%, respectively, which is due to the aging of the population and its increase in size [2, 4]. This highlights the need for further research aimed at identifying new biomarkers and therapeutic targets for this cancer type for its timely diagnosis and selection of effective therapy.

Technologies for analyzing the transcriptome profile of tumor cells made it possible to identify the so-called genetic signatures—specific sets of genes whose expression level in tumor tissue is measured and used to assess the risk of relapse and sensitivity to therapy [5]. The use of such genetic signatures makes it possible to identify patients at high risk of developing distant metastases already at early stages of the disease, which facilitates the prescription of more effective treatment regimens and improves prognosis [6].

Earlier, using a new approach to identifying prognostic biomarkers, our laboratory developed a gene classifier that makes it possible to assess the risk of breast cancer recurrence [7]. Two genes, *ELOVL*5 and *IGFBP*6, demonstrated the greatest prognostic value; low expression levels of these genes were associated with an unfavorable prognosis. The prognostic significance of the classifier was confirmed by further studies. In particular, it was shown that a decrease in the *ELOVL*5 expression increases the metastatic potential of tumor cells [8].

*ELOVL*5 is an elongase, which is involved in the elongation of long-chain polyunsaturated fatty acids (LCPUFAs), primarily omega-3 and omega-6, and is located in the endoplasmic reticulum membrane [9, 10]. In addition, this enzyme plays an important role in the synthesis of monounsaturated fatty acids [11]. It is noteworthy that the results of a number of studies in recent years indicate the significance of *ELOVL*5 gene expression in the development of other malignant neoplasms, in particular, prostate cancer and colorectal cancer [12–14]. However, it should be noted that the majority of modern studies focus not so much on the enzyme itself, but on the products of its catalytic activity (long-chain omega-3 and omega-6 PUFAs) and their impact on tumor cell processes or patient condition. For example, it was established that the presence of omega-3 LCPUFAs in the culture medium leads to a decrease in proliferation, migration activity, and invasiveness of tumor cells [15, 16]. In particular, the effect of these fatty acids can be exerted, among other things, via the induction of ferroptosis, an alternative mechanism of programmed cell death [17, 18].

Ferroptosis is an iron-dependent cell death pathway that differs from apoptosis and autophagy in morphological features and is characterized by the accumulation of lipid reactive oxygen species [19]. LCPUFAs play a key role in this process, inducing peroxidation of membrane phospholipids, which leads to disruption of cellular integrity [20]. Regulation of ferroptosis involves several mechanisms associated with lipid metabolism, iron homeostasis, activity of the amino acid antiporter of the Xc^−^ system (SLC7A11 and SLC3A2), involved in the metabolic pathway of glutathione biosynthesis, which is involved in the antioxidant defense of cells, as well as with the function of the mevalonate pathway and mitochondrial VDACs channels [21, 22].

One of the most studied ferroptosis inducers is erastin, which inhibits the SLC7A11 subunit and disrupts cystine transport into cells. This leads to a decrease in the level of glutathione and activity of GPx4, a key antioxidant enzyme, which contributes to the accumulation of reactive oxygen species (ROS) and promoted the induction of ferroptosis [22, 23]. Another target of erastin is the mitochondrial VDAC2/3 channels; disruption of their function increases ROS production and leads to mitochondrial dysfunction [22, 24].

Since tumor cells can often be resistant to apoptosis, but at the same time remain sensitive to alternative forms of cell death, such as ferroptosis, induction of the latter is considered a promising direction in antitumor therapy [25]. As mentioned earlier, LCPUFAs, including docosahexaenoic acid (DHA), can activate the ferroptotic pathway of cell death [17]. However, according to a number of studies, the mechanism of DHA-induced ferroptosis may differ from that triggered by classical inducers. For example, it was shown that, in breast cancer cells with knockdown of another prognostically significant gene, *IGFBP*6, ferroptosis is accompanied by less pronounced lipid peroxidation of mitochondrial membrane compared to that induced by erastin [26]. Interestingly, our previous study showed that *ELOVL*5 knockdown in breast cancer cells increases their sensitivity to LCPUFAs, with the observed cell death proceeding by the ferroptotic pathway [27]. Moreover, the results of the study of cell death kinetics, as well as transcriptome analysis, showed that a decrease in the *ELOVL*5 expression increases the sensitivity of cells to erastin-induced ferroptosis [28]. However, the specific features of ferroptosis induction by DHA in BC cells with suppressed *ELOVL*5 expression at the cellular level remain poorly understood.

The aim of this study was to perform a comparative analysis of the molecular response of sh-*ELOVL*5 breast cancer cells and control sh-LUC cells to DHA and the classic ferroptosis inducer erastin. In this study, we measured cell death dynamics and performed targeted assessment of expression of selected groups of genes involved in the key biological processes, such as regulation of ferroptosis (*GPX*4, *GCLM*, *SLC*7*A*11, *HMOX1*, *FTL*, and *SAT*1) and inflammatory response (*CXCL*8 and *IL*6), as well as control of cell cycle and proliferation (*CENPE*, *CENPI*, *CCNB*2, *ASPM*, and *KNL*1).

## MATERIALS AND METHODS

In this study, the MDA-MB-231 cell line was used as an in vitro model of breast cancer. The cell line with a stable knockdown of the *ELOVL*5 gene (sh-*ELOVL*5) was obtained previously using RNA interference [29, 30]. To obtain control MDA-MB-231 (sh-*LUC*) cells, we used the same lentiviral vector pLVX shRNA1 containing shRNA to the *Photinus pyralis* luciferase gene. MDA-MB-231 breast cancer cells sh-*LUC* and sh-*ELOVL*5 were cultured in DMEM high-glucose medium (Gibco, United States) supplemented with 10% FBS (Capricorn, Germany), 1% Glutamax (Gibco, United States), and 1% Anti-anti (Gibco, United States) at 37°C and 5% CO_2_ in a cell incubator (Alphavita, China). Cell cultures were passivated with 0.25% trypsin–EDTA solution (PanEco, Russia) every 2–3 days. Cell growth dynamics were assessed visually using a ZOE Fluorescent Cell Imager inverted microscope (Bio-Rad, United States).

To analyze the kinetics of ferroptosis, cells were plated in a 96-well plate at a density of 10 000 cells per well and incubated in 100 μL of culture medium in a cell incubator (37°C, 5% CO_2_) for 24 h. The culture medium was then replaced with the control medium or a medium containing 5 μM erastin, 200 μM DHA (Sigma-Aldrich, United States), 100 μM deferoxamine (DFO, Acros Organics, United States), or 0.5 μM ferrostatin-1 (Sigma-Aldrich, United States), as well as their combinations. The cells were incubated at 37°C and 5% CO_2_ for 24 h. To visualize dead cells, the added medium also contained propidium iodide (PI, Lumiprobe, Russia) at a concentration of 1 μg/mL. Since the red fluorescent signal could only be detected after cell membrane disruption, the cell death kinetics were determined by counting the number of red fluorescent objects (dead cells) over time using the IncuCyte® S3 vital cell assay system (Sartorius, United States).

To analyze viability after analyzing the cell death kinetics, an MTT assay was performed. For this purpose, the medium in the wells of the plate was replaced with a medium containing a 10% MTT solution (PanEco, Russia) in DPBS (PanEco, Russia) at a concentration of 5 mg/mL, and the plates were incubated for 2 h. Then, the cells were lysed with a solution containing 10% sodium dodecyl sulfate, 50% dimethylformamide, and acetic acid (pH 4.7) [31]. The treatment of cells with the drugs and their combinations was performed in triplicate. Analysis of variance (ANOVA) was used to assess the statistical significance of the differences observed.

Quantitative real-time PCR (qPCR) was used to analyze changes in the expression of the genes involved in the regulation of ferroptosis. MDA-MB-231 sh-*LUC* and sh-*ELOVL*5 cells were plated at a density of 50 000 cells per well in 24-well plates and incubated for 24 h at 37°C and 5% CO_2_. The medium was then replaced with a control medium or a medium containing 4 μM erastin or 200 μM DHA. After 24 h, the cells were lysed, and RNA was extracted using the SKYprep RNA Pure Micro Kit (SkyGen, Russia) according to the manufacturer’s recommendations. cDNA synthesis was performed using the MMLV RT Kit (Evrogen, Russia).

qPCR reactions were performed using qPCRmix-HS SYBR dye (Evrogen, Russia). Oligonucleotide primers were selected based on sequences from the UCSC Genome Browser using Primer-BLAST. Their specificity and absence of dimerization were verified using OligoAnalyzer 3.1. *ACTB* was used as the reference gene. Primer sequences are presented in Table 1. Statistical significance was assessed using a two-way ANOVA with Tukey’s test. All experiments were performed in triplicate.

**Table 1.** Sequences of oligonucleotide primers for qPCR

Gene	Sequence
*ACTB*	Forward 5'-CTGGAACGGTGAAGGTGACA-3'
	Reverse 5'-AAGGGACTTCCTGTAACAACGCA-3'
*ANGPTL*4	Forward 5'-CAAGCCTGCCGAAGAAAGA-3'
	Reverse 5'-GTTGAAGTCCACTGAGCCATC-3'
*ASPM*	Forward 5'-ACAAAACCCATTATCGCTGTGGCA-3'
	Reverse 5'-GCATCGGGTGTCTGGGAATATCTGT-3'
*CCNB*2	Forward 5'-CTGCTTCCTGCTTGTCTCAGAAGG-3'
	Reverse 5'-AGGCAAGGTCTTTGACGGCTTTT-3'
*CENPE*	Forward 5'-AACACTTACTGCCTCTCCAGTTTGCC-3'
	Reverse 5'-TTCCATTGCCTGAGCCCGC-3'
*CENPI*	Forward 5'-AACATCCATTGCTTAGAGCTGCCTT-3'
	Reverse 5'-AGCACTCTGCCCTGATGATGGT-3'
*CXCL*8	Forward 5'-AGGAACCATCTCACTGTGTGT-3'
	Reverse 5'-TTGGCAAAACTGCACCTTCACA-3'
*GCLM*	Forward 5'-AGCATTTACAGCCTTACTGGGAGGA-3'
	Reverse 5'-TGGTTACTATTTGGTTTTACCTGTGCCC-3'
*GPX*4	Forward 5'-CGCCCGATACGCTGAGTGTG-3'
	Reverse 5'-TGGCTCCTGCTTCCCGAACT-3'
*FTL*	Forward 5'-CGCCAGAACTACCACCAGGAC-3'
	Reverse 5'-CCCGCTCTCCCAGTCATCAC-3'
*HMOX*1	Forward 5'-TCAAGCAGCTCTACCGCTCCC-3'
	Reverse 5'-TTGGTGTCATGGGTCAGCAGC-3'
*IL*6	Forward 5'-TAACCACCCCTGACCCAACCA-3'
	Reverse 5'-GTGCCCATGCTACATTTGCCGAA-3'
*KNL*1	Forward 5'-GGACCCTCTGGACTTCAGCACTTA-3'
	Reverse 5'-GCCTGGGAGATTGCTCTGTCG-3'
*SAT*1	Forward 5'-GTGCCGAAAGAGCACTGGACTC-3'
	Reverse 5'-TGCCAATCCACGGGTCATAGGT-3'
*SLC*7*A*11	Forward5'-CCGCAAGCACACTCCTCTACC-3'
	Reverse 5'-CAAAGCTGGGATGAACAGTGGCA-3'

## RESULTS AND DISCUSSION

**Fig. 1.**
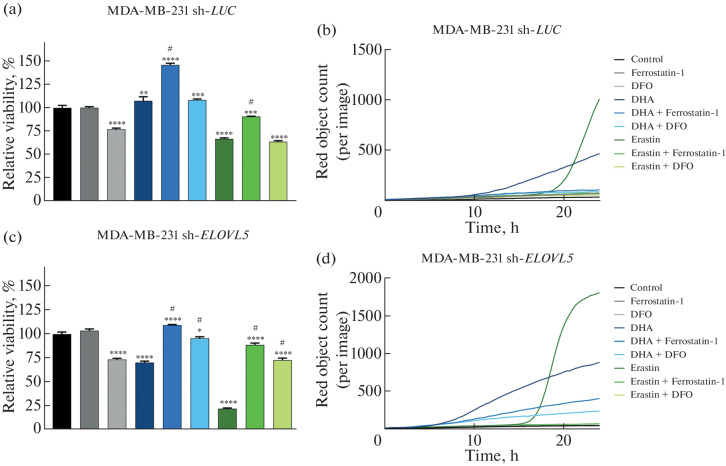
Results of the evaluation of the viability of (a, b) MDA-MB-231 sh-*LUC* cells and (c, d) sh-*ELOVL5* cells after 24-h incubation with DHA (200 μM), erastin (5 μM), ferroptosis inhibitors ferrostatin-1 (0.5 μM) and deferoxamine (100 μM), as well as their combinations using the MTT and IncuCyte assay. * Statistically significant difference compared to the control group (without treatment); * *p*-value < 0.05; ** *p-*value < 0.01; *** *p*-value < 0.001, **** *p*-value < 0.0001; ^#^statistically significant difference compared to the group treated with DHA or erastin only.

On the basis on the MTT assay results (Figs. 1a, 1c), the effects of docosahexaenoic acid (DHA) and erastin on the viability of MDA-MB-231 sh-*ELOVL*5 cells and the control sh-*LUC* cells were assessed. Additionally, the possibility to inhibit the DHA- or erastin-induced cell death was studied using the known ferroptosis inhibitors—ferrostatin-1 and deferoxamine (DFO). DHA and erastin concentrations, as well as incubation times, were selected on the basis of the results of preliminary titration and real-time cell death analysis using IncuCyte so that the cytotoxic effects were clear while maintaining a significant proportion of viable cells for assessing changes in the expression of individual genes using qPCR.

According to the data obtained, the treatment of both cell lines with erastin resulted in a significant decrease in their viability, while the addition of the classical ferroptosis inhibitor, ferrostatin-1, promoted restoration of viability in both control cells (sh-*LUC*) and sh-*ELOVL*5 cells (Fig. 1). However, deferoxamine (DFO) had no noticeable protective effect against erastin in the case of sh-*LUC*. In contrast, in the case of cells with the *ELOVL*5 gene knockdown, the addition of DFO resulted in a significant increase in viability. This difference in the effect of DFO may be due to the fact that the decrease in the viability of control sh-*LUC* cells caused by erastin is quite small and comparable to the effect of DFO itself. It should be noted that, according to IncuCyte data, no significant cell death was observed during incubation with DFO, and it also completely prevented cell death from erastin (Figs. 1b, 1d). Since the MTT method reflects not only death but also general metabolic activity, the decrease in absorption caused by DFO may be due to both a deceleration of proliferation and metabolic restructuring.

Interestingly, incubation with DHA, conversely, had different effects depending on the cell line. In the control cells (sh-*LUC*), DHA did not significantly alter viability compared to the control. However, in sh-*ELOVL*5 cells, a decrease in viability to approximately 75% of the control level was observed. These results indicate an increased sensitivity of sh-*ELOVL*5 cells to LCPUFAs. The fact that DHA induces ferroptosis in sh-*ELOVL*5 cells is confirmed by a significant recovery of viability upon the addition of ferrostatin-1 and deferoxamine. It should be noted that, while the protective effect of ferrostatin has already been demonstrated previously, the use of deferoxamine for the first time additionally confirmed the ferroptotic mechanism of cell death in these cells in this case.

To study the dynamics of ferroptosis, the IncuCyte intravital cell assay system was used. The results of the analysis demonstrated that the cytotoxic effects of both drugs on sh-*ELOVL5* cells were more pronounced compared to those in the control sh-*LUC* line (Figs. 1b, 1d). This is evidenced by a greater number of fluorescent red objects (cells with disrupted membrane integrity) in the case of sh-*ELOVL*5 compared to the control cells. In addition, we noted that sh-*LUC* cells are apparently more resistant to the treatment than sh-*ELOVL*5, as evidenced by the difference in the time of cell death. For example, the toxic effect of the drugs on the control cells manifested itself 2–3 h later than in the case of the sh-*ELOVL*5 cells. This observation is consistent with our previously published data obtained for erastin based on the analysis of the dynamics of confluence changes, without the use of the fluorescent dye [28]. It is also noteworthy that cell death of both the control and sh-*ELOVL*5 cells in the presence of DHA occurred earlier than in the presence of erastin. In particular, ferroptosis induction in sh-*ELOVL*5 cells by DHA was observed 8 h after treatment, whereas in the canonical inducer erastin it was observed 10 h later. *ELOVL*5 knockdown is known to cause critical changes in cellular lipid metabolism. Im particular, impaired accumulation of lipid droplets, which are required for storing free fatty acids and preventing their peroxidation, was demonstrated in *ELOVL*5 knockdown cells, as well as an accelerated uptake of LCPUFAs from the culture medium by these cells due to their intracellular deficiency. All this makes sh-*ELOVL*5 cells more sensitive to the external effects of LCPUFAs, such as DHA, as well as to the induction of ferroptosis [27].

Addition of the ferroptosis inhibitors ferrostatin-1 and deferoxamine in combination with both DHA and erastin resulted in a significant reduction in cell death in both cell lines, confirming that ferroptosis is induced by both compounds. Interestingly, in sh-*ELOVL*5 cells, the addition of ferroptosis inhibitors in combination with DHA resulted in only partial suppression of cell death. However, a combined use of the same inhibitors with erastin completely prevented ferroptosis, and cell viability remained comparable to the control. These data suggest differences in the molecular mechanism of ferroptosis induced by erastin and DHA in MDA-MB-231 sh-*ELOVL*5 cells compared to the control cells.

Then, to better understand the specifics of intracellular molecular processes, a comparative analysis of changes in the expression of individual genes was performed using qPCR in the control cell line and in sh-*ELOVL*5 cells. We studied the genes that were directly involved in ferroptosis. For example, the classic targets of the cellular stress transcription factor Nrf2, such as *HMOX*1, *SLC7A*11, *FTL*, and *GCLM* were analyzed [32]. We also analyzed the expression of *GPX*4, the key gene involved in defense against ferroptosis. These genes play a key role in maintaining redox homeostasis, regulation of iron metabolism, and protection against oxidative stress during ferroptosis [33].

Modern studies have shown that ferroptosis is often accompanied by the development of an inflammatory response [34]. For example, ferroptosis results in the release of proinflammatory molecules such as interleukins (IL)-1β and IL-18 [35]. In addition, in recent years, more and more studies demonstrate the significant benefits of ferroptosis inhibitors in the treatment of inflammation [36, 37]. It is worth noting that DHA is generally considered an antiinflammatory molecule that reduces the activation of the NF-κB signaling pathway and the production of cellular markers of inflammation [38, 39]. However, the specific feature of inflammatory processes in sh-*ELOVL*5 cells treated with DHA remain poorly understood. For this reason, in addition to classical markers involved in ferroptosis regulation, in this study we also analyzed the genes associated with inflammation, including *CXCL*8 (*IL*8) and *IL*6, which encode the key proinflammatory cytokines. In addition, to assess the potential effect of DHA and erastin on cell proliferative activity and cell cycle progression, the genes regulating mitosis and cell division (*CENPE*, *CENPI*, *CCNB*2, *ASPM*, and *KNL*1) were also included in the study. This approach allows for a comprehensive comparative analysis of the responses of the studied cell lines to various ferroptosis inducers.

**Fig. 2.**
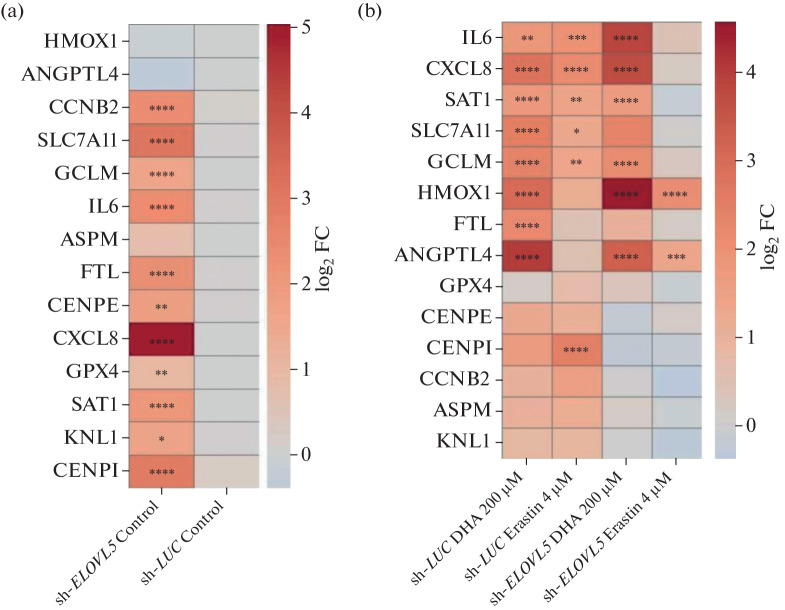
Heat map comparing the expression of selected genes in MDA-MB-231 sh-*LUC* and sh-*ELOVL*5 cell lines (a) without treatment with drugs and (b) after treatment with 200 μM docosahexaenoic acid or 4 μM erastin compared to the control (without treatment) based on the real-time qPCR. * *p*-value < 0.05; ** *p*-value < 0.01; *** *p*-value < 0.001, **** *p*-value < 0.0001.

The expression analysis of the selected key genes revealed significant differences of the MDA-MB-231 sh-*ELOVL*5 cells from the control cells (sh-*LUC*) (Fig. 2a). In particular, a significant increase in the expression of the proinflammatory cytokines *CXCL*8 (*IL*8) and *IL*6 was observed, indicating activation of inflammatory signaling cascades in sh-*ELOVL*5 cells. This inflammatory state was apparently associated with disturbed lipid metabolism due to decreased activity of *ELOVL*5, which is responsible for the synthesis of polyunsaturated fatty acids, including ω-3 docosahexaenoic acid and eicosapentaenoic acid [9, 10]. Their deficiency can promote the activation of proinflammatory cascades due to lipid imbalance and reduced synthesis of antiinflammatory compounds from PUFAs (resolvins and protectins).

In addition, in sh-*ELOVL*5 cells, we detected increased expression of several genes involved in the regulation of cell cycle and proliferation. For example, increased levels of *CCNB*2 (cyclin B2), which functions in a protein complex with *CDK*1, indicate an increased transition of cells into mitosis and activation of processes that ensure nuclear membrane breakdown, chromatin condensation, and mitotic spindle formation [40]. Similarly, increased expression of *CENPE* and *CENPI* (genes encoding centromeric proteins involved in the attachment of chromosomes to spindle microtubules) indicates increased cell proliferative activity [41, 42].

Additionally, in the sh-*ELOVL*5 cell line, activation of antioxidant system components was detected, including a significant increase in the expression of *GCLM* and *SLC*7*A*11 and a slight increase in the content of *GPX*4 mRNA, indicating changes in the functioning of the cellular defense system against oxidative stress [33]. It is noteworthy that this enhanced antioxidant defense in the *ELOVL*5-knockdown cells did not, however, result in an increased resistance to ferroptosis; on the contrary, these cells were more sensitive to it. This apparent contradiction can be explained by the simultaneous increase in the generation of reactive oxygen species due to putative mitochondrial dysfunction in the sh-*ELOVL*5 cell line under the influence of ferroptosis inducers [27], which apparently outweighs the additionally activated oxidative stress defense mechanisms.

Thus, according to our results, the sh-*ELOVL*5 cell line is characterized by complex molecular features, including increased inflammatory activity, enhanced proliferation, and activation of antioxidant defense mechanisms. These characteristics are likely due to disturbed fatty acid metabolism in *ELOVL*5 deficiency, which entails a reorganization of associated signaling pathways.

Next, we analyzed the molecular changes in the MDA-MB-231 sh-*ELOVL*5 cells and control sh-*LUC* cells in response to treatment with 200 μM DHA or 4 μM erastin (Fig. 2b). The obtained data indicate that the control sh-*LUC* cells exhibit pronounced transcriptional activation in response to both ferroptosis-inducing agents. In particular, they showed a significant increase in the expression of the proinflammatory cytokines *IL*6 and *CXCL*8, as well as the stress-associated genes *SAT1*, *GCLM*, and *SLC7A*11, both upon DHA and erastin treatment. However, interestingly, an increase in the expression of heme oxygenase-1 *HMOX1*, a reliable marker of oxidative stress response activation, was specific for the treatment with DHA [43]. This indicates that, in the control cells, DHA-induced ferroptosis is accompanied by both inflammatory and redox activation, whereas erastin apparently triggers cell death through a slightly different mechanism, with a less pronounced involvement of the oxidative stress response.

In the sh-E*LOVL*5 cell line, the picture significantly differed from that in sh-*LUC*. For example, on the one hand, the DHA-induced changes in the expression of *IL*6, *CXCL*8, *SAT*1, and *GCLM* were retained, with both the proinflammatory cytokines (*IL*6 and *CXCL*8) and *HMOX*1 being significantly more activated. However, the transcription level of these genes in sh-*ELOVL*5 in response to erastin remained virtually unchanged. This may likely be due to the already high expression levels of these genes in the control. It is also worth noting that DHA activated inflammatory processes in both cell lines, which is in stark contrast to its previously described antiinflammatory effect [38, 39]. Apparently, this effect is due to the high concentration of DHA, which is at the upper limit of the physiological range in blood plasma and can trigger ferroptosis and associated inflammation.

In addition, the sh-*ELOVL*5 cells showed an increased *HMOX*1 expression in response to both ferroptosis inducers, whereas in the control sh-*LUC* cell line, this oxidative stress marker was activated only by DHA. This may indicate a reduced induction of oxidative stress in sh-*ELOVL*5 cells. Interestingly, other protective genes, conversely, were activated less strongly in sh-*ELOVL*5 cells upon treatment with erastin.

Interestingly, the expression of the vast majority of genes related to cell cycle regulation and proliferation (*CENPE*, *CENPI*, *CCNB*2, *ASPM*, and *KNL*1) showed statistically nonsignificant changes in both cell lines. This suggests that treatment with either DHA or erastin, at the concentrations and time points used, does not significantly affect cell proliferative activity.

Overall, DHA induced a more pronounced response, including the activation of both proinflammatory and redox-associated genes, whereas erastin predominantly induced an antioxidant response. *ELOVL*5 knockdown is apparently accompanied by an already elevated level of oxidative stress, which may limit the potential for further activation of defensive pathways by ferroptosis-inducing compounds and explain the increased sensitivity of the cells to erastin. At the same time, *ELOVL*5 expression deficiency results in a more pronounced response to DHA, though similar in terms of the activated genes to that observed in the control cells. Thus, the obtained data may open a promising direction for further in vivo studies and validation of the described effect of the *ELOVL*5 gene on the regulation of ferroptosis, since limitations of in vitro models such as the lack of reproducibility of tumor heterogeneity and microenvironment, as well as the influence of the immune system and hormonal status of the patient, do not allow for a reliable extrapolation of the present results to the clinical level.
